# Exploring educational and social inequality through the polyphonic voices of the poor: A *habitus listening guide* for the analysis of family-schooling relations

**DOI:** 10.1080/03050068.2018.1535644

**Published:** 2018-11-13

**Authors:** Arif Naveed, Madeleine Arnot

**Affiliations:** Faculty of Education, University of Cambridge, England, UK

**Keywords:** Habitus listening guide, comparative narrative research, rural Pakistan, gender, family research, social inequality, qualitative data analysis, voices of the poor

## Abstract

The aim of this methodological article is to contribute a new form of qualitative data analysis that is relevant for the comparative study of family cultures and schooling. We describe the development of our Habitus Listening Guide linking Bourdieu’s theory of social reproduction to critical narrative theory. The interpretative tool outlines (a) social-structural (b) horizontal intergenerational (c) vertical gender and (d) mythic-ritual listenings which can be used to explore the engagement of youth and their families with schooling. Such listenings reveal the dispositional positioning of schooling in family values and the complex structural and human relational effects of schooling on family members’ livelihood and wellbeing. It offers the possibility of comparing families in terms of their gendered and generational relations and the ways in which religious and mythic-ritual discourses legitimate their aspirations in the context of changing communities. The Guide offers a way of accessing and comparing subjective micro level experiences of social inequality and the contribution that schooling plays, or is expected to play, in relation to individual and/or family social mobility.

## Introduction

Over the last ten years, comparative studies of education have been alerted to concerns about the use of metropolitan methodological traditions, especially if they reinforce a highly individualised Western approach to the study of social relations and demonstrate a lack of reference to different ways of being in the Global South. Takayama, Sriprakash, and Connell (2016, S1) in a seminal paper, have argued that there needs to be a ‘major rethinking’ by comparative and international educationalists ‘about the norms and knowledge about difference, comparison, and research that have been inherited from the field’s history’. These authors quote Ninnes and Burnett (2004, 196) who argued that the engagement of comparative education with an ‘Other’, on the whole, ‘has not been problematized’, and point out neither has the ‘critical role that uneven power relations play in the constitution of its comparative knowledge’ been sufficiently challenged (op cit. S3)^1^. According to Arnove (2001), it is yet possible to expand the boundaries of the comparative education field, especially if there is more engagement with different regions of world. But arguably this will involve, following Smith (2012), developing a ‘decolonising methodological agenda’. In light of these challenges, we put forward a different approach to hearing the voices of ‘Others’ – those whose lives in poverty are greatly disadvantaged by precisely such ‘uneven power relations’^2^ especially when represented through homogenising, pathologising or victimising discourses. The diversity of the educational experiences, aspirations and practices of those living in poverty belie the universalising representation of the ‘poor family’ and the ‘poor child’ (Hopkins and Sriprakash 2015) or the ‘third world woman’ (see Fennell and Arnot 2008).

Our primary aim here is to outline how we developed a form of narrative analysis that opens up an alternative view of the poor, whether in high, middle or low income countries, where access to schooling for such communities (particularly, but not only, for girls) is thought to be massively constrained by ‘traditional’ family cultures. Despite an increasing interest in international education and development studies to capture the role of families and households in relation to school choice, school achievement, social mobility and poverty alleviation (see Camfield and Roelen 2013; Kim 2017), the methodological challenges are not yet resolved. Our article contributes to this particular agenda by finding a sociologically informed way to uncover the valuing and experiences of schooling by family members.

The model of qualitative data analysis we have developed brings together the insights from comparative education and development studies, contemporary narrative theory, and Bourdieu’s (1977) anthropologically-inspired notion of the *habitus* that tries to capture the deep structural and emotional forces which shape the cultural reproduction of social inequality. We have drawn on such theoretical/methodological traditions to develop what we call a *Habitus Listening Guide* which facilitates the uncovering of families’ engagements with social and economic inequalities and with the unequal outcomes of formal schooling. By using such a guide, comparative educationalists will hear the complex poly-vocal voices of those living in absolute or relative poverty and will deepen their understanding of the intergenerational and gendered social (im)mobilities in poor communities.

With these goals in mind, we describe below our theoretical and methodological approach, how we designed and conducted our family case studies and how we interpreted our engagement with the ‘voices of the poor’. We then outline the ways we developed the theoretical framings for two early versions of the Guide, before describing each of four key listenings of voice data. We conclude by mapping the objectives and methods involved in using these listenings and the potential value of this approach for comparative/international education.

## Our theoretical and methodological approach

Our starting point is the family-centered design of a major research programme we conducted in rural Pakistan^3^ which purposively challenged individualised research strategies that are often used to tap the effectiveness of schooling for youth, particularly in Western societies. We took the view that, if a young person is treated as an individualised voiced subject, simply as someone in transition from home through school to work, then only limited explanations for the ensuing patterns (for example, the cost of schooling, cultural barriers and a lack of personal motivation) are likely to be offered. Such individualised models are likely to be embedded in modes of data analysis that track linearity rather than the continuing interchangeable movement of family members across statuses, types of socialisation and social roles (see Arnot et al. 2012). If educational research, internationally, is to understand issues around unequal access, quality and outcomes of schooling, it requires a good deal more sensitivity to local cultural environments, and the intimate gendered dynamics and collective religious, ethnic and other social identifications within families. With such knowledge, we begin to recognise and value the diverse identities *within* the category of ‘the girl child’ (Fennell and Arnot 2008) and the different masculinities at play in non-Western cultures. We would be reminded that young people in Southern contexts also daily, negotiate relational dispositions, ambitions and values which, in turn, shape their schooling choices and experiences and employment patterns. Such negotiations, particularly in rural cultures, are strongly influenced by extended families and their positioning within local social orders.

The approach we have developed gives researchers greater purchase on such family dynamics but also on the forms of family change associated with the ‘splash-back effect’ of mass schooling on young people. Our research builds on the tradition of those in comparative and international education who believe that deeper family narratives have a key role to play. This tradition is shaped by an interest in the role of life and oral histories and in-depth semi-structured or open-ended interviews in family-schooling research (Slim and Thompson 1993; Davis 2006). Hulme (2004), for example, pointed to the value of a one family case study in which he reconstructs the social dynamics in the life stories of Maymana (the mother) and Mofizul (her son), illustrating how they slid into and endured poverty, and how they explained it. The detailed accounts of this mother and son suggest significant roles for key institutions including state, market, and civil society. This single case study highlights the importance of exploring gender, age, and disability dynamics, the limited demand for the labour in poor areas and, at broader level, the constrained participation of the poor in markets (given the unequal social structure), the lack of health services, and the weak outreach of various institutions. Hulme recommends therefore that we need to ‘think small’, through what he calls ‘nano-level’ research, in order to counterbalance the big ideas of poverty reduction programmes. Research in developing countries relies often upon macro-level information which constructs ‘average’ poor people or households in order to design programmes, yet research at the micro-level reveals family members, relatives and neighbours as ‘key agents in the process that reduce (and sometimes create) human deprivation’ (Hulme 2004, 162).

Family case study research can, as Davis (2006) also found, reveal patterns of crisis, coping strategies and opportunities of those living under poverty. His analysis of 90 life history interviews allowed him to classify three different types of household trajectories which he variously described as ‘*stagnant’, ‘improving’* and ‘*declining*’ which could also be ‘*smooth*’, ‘*saw-tooth’*, ‘*single-step*’ and ‘*multi-step’*. Using this typology, Davis reported that improvements in the lives of people happen only gradually whilst various crises, such as health shocks, lead to sudden declines. Families also bear witnesses to considerable economic and social change, some of which point to the value of schooling as a family enterprise whilst other changes indicate growing new social inequalities between the educated and the oft-called ‘uneducated’ (Arnot and Naveed 2014). In this microsystem, families are key decision-makers – a fact recognised by education programme developers, even if their understanding of the roles that families play is scanty or assumed to be similar to the role of families in developed contexts (Kim 2017, 3).

However, Sung won Kim’s (2017) recent review^4^ of studies of parental-school involvement in major international education journals including *Comparative Education* exposes the paucity of family research in those developing countries which are trying to achieve universal education even though ‘there is a real need to understand how parents can uniquely contribute to children’s education in developing countries and to identify how they are similar or different from developed contexts’ (3). The notable search for deeper understanding through the study of individual households rather than children is the focus of the *Young Lives* project^5^ which explores why some households remain poor, some became poor and some move out of but drop back into poverty as a result of various ‘drivers and maintainers’. Camfield and Roelen (2013)’s study of Ethiopia within this project argues that the value of qualitative case studies (QSAs) lies in offering a variable-centred approach with which to compare different households; they add value because they provide a longitudinal account of the effects of particular events, and more importantly the effects on households’ responses to them (596). Below we describe how, in our international project, we ourselves engaged with this challenge of working with qualitative family case studies.

## Collecting family-centered data on educational outcomes

In 2005, we designed and ran a family-centred study of educational outcomes in Ghana, India, Kenya and Pakistan. In each country we used a stratified sample of 10 households in both a rural and an urban setting, in which the one son and one daughter (each aged between 15 and 25 with either no schooling, only basic schooling or post-basic schooling) were sampled along with their biological mother and father or their main male/female adult carers. The parent/carers were randomly distributed across the different schooling levels. The striking feature of our research was that none of the families had identical educational profiles or trajectories (Arnot and Naveed 2014) – a fact that belies any attempt to universalise the educational levels or family cultures of poor families. Using in-depth taped interviews in their own language (Punjabi), we captured rich data on how the role of schooling in the two Punjab sites was perceived, valued, and how it affected the lives of youth and their parents/carers. We asked each of the four household members about intended/unintended, actual and desired outcomes of schooling for themselves and their children or, in the case of young people, what they desired for their future children.

When collecting narrative qualitative data in this fashion, we were aware of our own positioning and that of the research team. As far as possible we ensured that all interview teams came from the region, spoke the local language and were trained to conduct ethical, consensual interviews in a manner that was sensitive to local cultures, hierarchies, customs, and gender concerns. However, rarely did family members have the educational levels of the interviewers. The stories told by respondents would have been affected by the interviewers’ age, gender and education and their own status in the community, how the former viewed the importance of the interview and whether their ‘tellings’ would make any difference to their family’s lives. During the course of the project, we conducted a small meta-study by interviewing the researchers to help us understand the context (time, place, access, levels of trust, gender and language) in which the actual interviews were conducted.^6^ When analyzing the data, we took into account that these taped interviews were forms of self-representation which were shaped by this context.

Reading the rich literature on the nature of such narrative data, we were alerted to the polyphonic, often contradictory, nature of such voices and the multiple layers of interpretations that are filtered through various hierarchies (Brown and Gilligan 1992; Abbott 2008; Horsdale 2011). The four interviews within one family quickly revealed to us that within the relational environment of the home lie neither one family voice, nor just one person’s view. Families and individual members are polyphonic, as they speak in the languages, for example, of their caste, class, culture, religion, gender, sexuality and disability (Singal 2011). Doucet and Mauthner (2008), who draw on Stanley’s (1993) insights, argue that narrated subjects are ‘not constituted in language or discourse but are constituted in relation to other subjects and to the ‘material reality of everyday life’’ (403). They believe that, although a subject knows and experiences, ‘we cannot … fully know that subject’:

… there may well be something ‘beneath’ or ‘behind’ or outside narrative; nevertheless, all we can know is what is narrated by subjects, as well as our interpretation of their stories within the wider web of social and structural relations from which narrated subjects speak. (Doucet and Mauthner 2008, 404)

We therefore needed to find a practical way of getting behind the narrative to work ‘reflexively with both critical and constructed subjects and with translating epistemological conceptions of relational narrated subjects into research practice’ (Doucet and Mauthner 2008, 404).

Critical narrative analysis signals an important departure from the mainstream tradition of taking voices of the poor as ‘factual’ accounts or ‘true stories’ of their lives, or necessarily as emancipatory, empowering or ‘authentic’. As Horsdale (2011, 87) argues, the narrated accounts of our research participants ‘represent situated interpretations of life and experience’ – hence ‘fictional facts’ (Denzin 1989, 76) shaped by context, researcher, time, place and audience. Such fictional reconstructions of the relationship between various events, and between events and their social contexts are invaluable (Coffey and Atkinson 1996: cited in Bryman 2012, 584). Hence, we wanted to listen to how our interviewees, through their narratives, actively made sense of their world and the key events in their lives; how they constructed their ‘own place’ in a highly stratified and unequal social order which, in turn, shaped their strategies and practices – all of which were shifting continuously.

As part of our own reflexive process we needed to think about the process of ‘*telling’* beyond what is ‘told’ (Reissman 2008, 77). We needed to pay attention to how narrative structures and the relationships between various segments of family members’ narratives are affected by the context of narrative production such as the interview settings (Labov 1981; Reissman 2008, 91). Consequently, our listening needed to offer a reflective dialogic analysis of the narratives we tapped (Reissman 2008, 105). We needed to consider the audience of the speech and its purpose, and to bear in mind especially Bakhtin’s (1981) recognition of the multiplicity of voices and meanings within embedded power relations, and the historic and social continuity of each narrative:

Form and meaning emerge *between people* in social and historical particularity, in a dialogic environment. Every text, (…) [Bakhtin 1981] argues, includes many voices – hidden, internal politics, historical discourses, and ambiguities – beyond the author’s voice. Narratives (especially those that appropriate theatrical conventions) are polyphonic – multivoiced: the author (speaker) does not have the only word, that is, the authority over meaning is dispersed and embedded … . (Reissman 2008, 107) [our addition]

Consequently, instead of taking the narrator as the ‘final’ authority, we needed to ‘interrogate particular words, listen to voices of minor characters, identify hidden discourses [that] speakers take for granted, and locate gaps and indeterminate sections in personal narrative’ (Reissman 2008, 107). From this perspective, a narrative becomes a two-way dialogic act involving the narrator’s narration and the listener’s emotions. Each narrative *initiates* or *alludes* to other narratives generating a sense of what we call *inter-narrativity*.

With these strictures in mind, we saw our transcripts as offering only glimpses into what could be told about schooling, especially given the association that schooling might have with painful memories of denial or failure. The accounts of family members revealed how they saw schooling as part of financial decision-making, of the aspirational world, and of the hoped-for solution to the circumstances they found themselves in. They offered multiple stories with different interpretations about what school attendance was doing for them and to them. We asked family members to talk to us about how schooling had affected their work, citizenship and their sense of empowerment, and their strategies for self-protection (Arnot et al. 2012). However, family members also created their own agenda – choosing to talk about how their schooling was shaped by their own childhood dreams of learning and their current concerns about their place and caste/ethnic status in the community, their religious duties, and their concerns about government policy and their family’s future. Like others, we were humbled with the ‘inexhaustibility’ of the interview transcripts as we exposed ourselves to the dynamics of reading them (Iser 1974; 280 cited in Abbott 2008, 92) and struck by the complexity of the lives of the rural poor, their interconnectivity, and by the multiplicity of the meanings, possibilities and realizations contained within their voices (Abbott 2008).

Finally as Western-educated researchers of the cultures of ‘Others’ (in this case those living in poverty), we needed to understand the impact of our forms of data analysis – these narratives do not come to life unless we collaborate willingly (Abbott 2008, 86). Researchers bring forth the ‘voices’ of the poor as ‘contextually situated’ interpreters of such narratives (Horsdale 2011, 86) and in so doing we can tend to ‘overwork things that are there and put in things that are not there’. Horsdale argues, we may have a tendency to *underread* but also to *overread* (Horsdale 2011, 86). This insight is particularly relevant to studies of the poor whose narratives can remain largely *underheard* (a point we return to later). Little advice is offered on how to listen to data drawn from communal poverty shaped environments with strongly interconnected family members in ways that resolve such methodological problems. Below we describe how we evolved separately and jointly a *Habitus Listening Guide*, taking it through two versions before finalising its focus and functioning.

### Developing the habitus listening guide

Naveed’s (2013) first framing of a listening guide was inspired by Brown and Gilligan’s (1992) listening guide which draws upon clinical and literary approaches and relational theory. Their listening guide uncovered narratives about the interviewees’ sequence of events and named significant others, as well as their construction of what Brown and Gilligan called an ‘I-poem,’ and their relations with researchers. However, working in a rural Southern context suggested that more was needed in terms of exploring collective relational contexts where the ‘We’ as well as the individualised ‘I’ in Brown and Gilligan’s formulation, are equally relevant. Here Doucet and Mauthner’s (2008) and Pande’s (2014) different listening guides were important. Doucet and Mauthner’s first reading is entitled *relational and reflexively constituted narratives* whilst their second reading focuses on what they call *tracing narrated subjects* which *inter alia* looks at the social context that the narrator speaks about (406). They trace ‘the active ‘I’ who tells the story but also record shifts in narratives between ‘I’, ‘We’, and ‘You’, noting the varied meaning in the perception of the self and the ‘emerging narrated self’ (406). Their third *reading for relational narrated* subjects adds an exploration of social networks and intimate relationships – a narrated self-in-relation (a valuable concept for our Punjab study).

Of value too was the listening guide which Pande (2014) developed for her study of young Indian carers. Her listening guide had two new elements^7^. First, the use of three ‘contrapuntal’ themes (*school narratives, work narratives,* and *care narratives* (116)) which distinguished between the counterpoints and the multiple facets of each narrative, and traced the relationship between first person voice and the contrapuntal voices. Secondly, she established a *listening for the relational world* (117) when she explored key relationships such as immediate and extended family, friends and neighbours, this time emphasising the degree of emotional closeness – the distance – and hierarchy within these relationships (118). A major step in her analysis was to link these findings to the ways in which such relationships enable or constrain each person’s capabilities as defined by Sen (1999) and listed by Nussbaum (2000). Pande’s narrative analysis revealed the ways in which single representations of young carers living in poverty such as child labourer, school absentee or adolescent carer did not capture the multiplicity of meaning and interpretations in their complex agentic narratives.

There was a danger however in that such early listening guides could remain at a psychological level of interpretation, even if linked to the social context in which research subjects are located. Doucet and Mauthner’s fourth *reading for structured subjects* took this on board when they linked micro-level narratives with macro-level processes and structures, identifying the dominant ideologies and the structured power relations that frame a narrative (a theme that is particularly relevant to the study of poverty and inequality). These authors locate narrated subjects as ‘structurally located within grand or macro-level narratives’ (407) which,

… seek to ‘reconstruct and plot over time and space the ontological narratives and relationships of historical actors, the public and cultural narratives that inform their lives, and the crucial inter- section of these narratives with other relevant social forces’. (Somers 1994, 620 cited in Doucet and Mauthner, 406)

Attracted to the idea of developing a more sociologically informed listening guide that could relate ‘voice’ to social structural conditions and power relations, Naveed (2013) used Bourdieu’s (1977) seminal concept of *habitus* (defined as durable and transposable bodily dispositions, perceptions, aspirations, ambitions, interactions and strategies of individuals (87)) to enrich the first version of what he called *Habitus Listening Guide*. Operationalising this concept (Lahire 2003, 332; Reay 2004) is clearly not easy, not least because habitus remains inaccessible to individuals’ own thoughtful analysis (Bourdieu 1977, 183). Yet the project was eminently sensible if social injustice and inequality were to inform the mode of data analysis. The methodological challenge was to consider how to work *under* the level of consciousness, overturning ‘the conscious intentions’ of the actors (Bourdieu 2001, 79). Individuals’ subconscious processes affect their perceptions and strategies, making them ‘maintain separations, distances, and relations of order(ing)’, with the resultant effect of reproducing the entire system of hierarchy (Bourdieu 1996, 82). The challenge for comparative education researchers, especially in the context of rethinking how to work with the ‘Other’ is to recognise the value and significance of such deep dispositional educational worlds of young people and their families.

Naveed initially used two listenings to explore the views of the father, mother, a teenage son and daughter in one family case study. This first attempt captured the perceived effects of schooling on the community, various educational histories, and individual and family values and aspirations across age and gender as well as what each family member considered to be worth achieving, what was achieved and what could be achieved as a result of schooling. He noted that ‘it is possible to hear social structures and individuals’ agency, their tendencies and propensities, aspirations, values, as well as their bodily dispositions’ (Naveed 2013, 55).

The second run of data analysis published by Arnot and Naveed (2014) focused on what we called the *rural family habitus***.** We reformulated the *Habitus Listening Guide* so that it uncovered three types of interlinked educational and social dynamics: (a) *intergenerational educational dynamics* (the educational biographies of parents and youth) (b) *ongoing gender dynamics* and (c) *changing social dynamics of the rural field*. We employed these three mutually exclusive but interlinked listenings to differentiate the *rural family habitus* of three Punjabi families living in poverty. For example Rehmat, the butcher, and his wife Lalarukh family struggled to calibrate what they called ‘a new age of education’ in which families were pressured to send their children to school. In contrast, the family habitus of Akhter (the farmer) and his wife Kiran attempted to reconcile worldly and religious education whilst the family of Aslam, a local businessman, and his wife Kinza recognised that daughters needed schooling but that ‘education does not make all equal’. We became aware of the degree of difference in their understandings of the role of education, their inter-family negotiations about schooling and their different political and social frustrations with their family’s progress. In each case, there was evidence of both reproductive and transformative forces at work within families.

Using these three listenings, the educational dispositions of each member of such families living in poverty are inscribed by their past and present experiences within the objective conditions of their living (Bourdieu 1977, 76) – a world shaped by unequal social relationships. From this perspective, the objective conditions of living (such as rural poverty and the relationships and socialisation embedded deep in family and community histories) are not just context but shape individuals’ dispositions. Our interviews, in effect, captured individual family members’ internalisation of external structures and their generation of meaningful practices within the familial context – but not always in the same way (Bourdieu 1984, 466). The children, in turn, inherited those effects of parental internalisation and practice but also negotiated their consequences. Thus, social inequalities persisted not only because of any systematic institutional discrimination – they also resulted from the deep-seated power relations evident in the individual family members’ dispositions (McNay 1999, 77) and through their resulting perceptions and actions.

Our theoretical interpretation begs the question about whether there is indeed ‘one family habitus’.^8^ Atkinson (2011) reminded us that researchers need to listen carefully for signs of individual agency within families, to recognise a person’s creative potential, over and above their collective socialisation within families and communities, and not to slip too easily into collective notions of habitus. Bourdieu believed that there is a uniqueness in individuals’ habitus that resulted from their unique ‘historical’ experiences of early life, family upbringing, schooling and social life. Having said that, family socialisation is chronologically integrated (Bourdieu 1977, 87) such that the earlier habitus shapes later experiences and, in this context, familial relational worlds and schooling assume a critical role.

According to Bourdieu, schooling has the potential to transform the habitus acquired in the family since it is another context for the formation of habitus. If we are to listen and work with such educational transformations, we need to remember that ‘the academic mechanisms of aggregation and segregation’ can become ‘the hidden mediations through which social hegemony is achieved’ (Bourdieu 1996, 183). Education systems and schooling institutions which deliver such aggregation and segregation are part of the unequal social order which shapes the poverty of such families. Therefore, we might also want to hear how socialisation within the schooling environment itself restructures family members’ dispositions – as a group of individuals rather than as a unit. Bourdieu is clear that any (re)configuration of the habitus acquired in the school over that acquired in the family will provide space for improvisation and transformation.^9^ Hence we understand that the youth we interviewed need to be seen not merely as passive education actors carrying their familial upbringing. Their improvised habitus can influence and ‘act back’ on the shared values and relations within the familial context.

A number of issues now affected our thinking. We needed to improve the *Habitus Listening Guide* such that it more systemically and directly engaged with the perceived role that schooling plays in creating social change within families. We had learned that the rise of mass schooling in Pakistan was seen as restratifying rural communities (Arnot and Naveed 2014). This finding encouraged us to look more deeply into the plot and subplot(s) of such social change. We also now needed to focus more attention on the ‘narrative sequence’ (when narrators recall and interpret their memories, talking about ‘there and then’ in reference to ‘here and now’ (Horsdale 2011)). By highlighting overlaps, convergence and divergence in family members’ narratives which *initiate* and *allude* to other narratives, we could begin to achieve a sense of the inter-narrativity (in the sense of Abbott’s (2008) ‘inter-textuality’). With these goals in mind, we redesigned our *Habitus Listening Guide* to include four revised listenings which we have now entitled:

1. A social structural listening
2. A horizontal intergenerational listening
3. A vertical gender listening
4. A mythic-ritual listening

**1. *A social structural listening***

This listening quite clearly now focuses on the social structural context in which families live, often in poverty. In a broad sense, we are tapping what Bourdieu and Wacquant (1992, 108–109) called a *field* which refers to the structure of relative positions within which the family is situated and its members think, act and take positions by virtue of the volume and structure of their capital, whether economic, social or cultural (Hilgers and Manges 2015). It is the field which contains interlocking, relative positions and which provides a context to understand the ways in which the household and its members ‘evolve or seek to evolve’, whether reproducing or transforming their positions (Hilgers and Manges 2015).

To tap the family-field relation, our first priority was to *underread* the transcript by excluding many details, perspectives, thoughts and emotions, with what Abbott (2008) calls a desire to ‘restore normality’ in family members’ disturbingly complex narratives, looking for a ‘single creative sensibility’ or a set of creative sensibilities. Of importance here is the task of specifically hearing and reconstructing the different family members’ educational biographies/histories – that of the father, mother, the son and the daughter. Our aim is to engage with the overlapping yet distinctively different description of the family’s life events (particularly when trying to escape poverty), their educational events, and the relationships between various educational events (Abbott 2008).

We then listen for the narratives in this round of data as *distinct forms of discourse* (c.f. Chase 2005) that enables each narrator to order their past events in ways that are meaningful to them and to the context in which they were brought up. The family and individual members’ discourse signalled the way they themselves experienced the communities they were members of, how they were brought up and how their pattern of schooling could be understood in light of the family’s past and present. Such discursive framing of schooling reveals the values and practices of family relationships, experiences, aspirations and sorrows in their own lives, that, when extracted, illustrate chronological plots connecting different parts of the family discourse to form a unique whole (Chase 2005, 65). It is here that the notion of habitus is particularly relevant since it refers to the relationship between such individual narratives potentially revealing the emotional and aspirational educational dynamics that shape the lives of parents and their children.

The reconstruction of the educational biographies of the four household members also elicits the ways in which the past survives in the present and perpetuates itself into the future – how ‘history is turned into future’ (Bourdieu 1977). Using the data from this listening, we gain insights into how the poor, driven by their immediate insecurities, make choices that procure security in the short run but postpone their long-term development prospects (Wood 2003). The listening reveals continuity, determinism, reproduction and the traces of (conditioned and conditional) transformation as well as the circumstances of family members who are stratified and subordinated to external and internal structures of power (Bourdieu 1977, 91). Further, the reconstruction of four interlinked cross-generational educational biographies sheds light on the social and political organisation of the community, and the capturing of the opportunities of local elites to reproduce the oppressive production of the notion of the ‘poor family’. The educational resources, opportunities and attainments of family members reflect such external material pressures but also the ‘splash-back’ onto parental aspirations and that of their sons and daughters.

**2. *A horizontal intergenerational listening***

The development of our horizontal intergenerational listening aims to see how social structures are acted upon in daily life, and are spoken about by, in this case, two generations – the husband and wife as one pair, and the son and daughter pair as another. A richer family study would have included the other children in the family as sibling influences are equally important, as well as the two sets of grandparents particularly because of the key role they play in such extended Pakistani families.

Here we compare and contrast the ‘symbolic structures’ (Bourdieu 1984) or ‘mental structures’ which the two different generations mobilise in order to make sense of, and participate in the social order outlined in first listening. Bourdieu (1984, 467) argues that the knowledge that people have is not a ‘mere reflection of the real world’, it also shapes the real world. These hidden meanings of speech can only be extracted by listening in an open, grounded fashion that enables us to understand the vastness of the motifs, perspectives and emotions contained in interview transcripts. When listening and comparing the two paired transcripts carefully over and over again, we treat these data as time–space situated interpretations of life and experience, i.e. taking stock of the ‘stuff’ (Horsdale 2011, 96). For example, of value to those interested in the short, longer and long term impacts of mass schooling is the observation of narrative theorists that it is through narratives that people organise their understanding of time. The ‘capacity to represent an event, either in words or in some other way, is the key gift and it produces the building blocks out of which all the more complex forms are built’ (Horsdale 2011, 13). Our two generations (parents and young people) build *stories* – narratives that ‘unfold in time and cover a space of time’ (Horsdale 2011, 89). Such theory encourages us pay attention to the temporal sequence of important events in the lives of the poor (Bryman 2012, 582; Abbott 2008) – what Reissman (2008, 64) called ‘discursive constructs of the historical contingencies’.

We also listen to what is ‘told’ in the interviews about the role of schooling in improving lives by the parental generation, and then compare these data to those offered by the son and daughter, looking for the contents of their speech, ‘the acts’, and ‘the events and cognition to which language refers’, (Reissman 2008, 58) as well as the ‘moral of the story’ of such contents, acts and events (Reissman 2008, 62). Our approach is not to fracture the narrative, instead we try to keep the story ‘intact’ – each of the four family members’ entire transcript continues to be our unit of analysis, at the same time it is valuable to explore different educational motifs in their stories. For example, uncovering;

- educational dispositions including values and perceptions;
- aspirations – about life in general as well as specific to education;
- educational strategies in the wake of deprivation;
- attitudes towards gender, generational persistence and change;
- familial obligations and responsibilities; and,
- schooling and the reproduction of social inequality.

In practical terms, the strategy of this so-called *horizontal intergenerational* round of listening is to *pair-up* the narratives of the father and the mother, the parental generation, and listen to them together, one by one, individually and sequentially. We repeat the same by pairing the narratives of the participating son and the daughter and then compare the two sets. We particularly attend to the terms with which the two narratives, of husband and wife or son and daughter, speak to each other. In addition to the relationships present, established, maintained and challenged *within* these voices, there are relationships *between* their generational voices with the effect that the four members of the same family co-construct a collective narrative about education. We, therefore, bring the narratives of the family members into a *dialogue, speaking to us*, and to *each other*, providing glimpses of the interacting worldviews. Sometimes there is a convergence of generational voices, at other times, each individual retains a distinctive voice and diverges from or even challenges that of other in their generation or, jointly across generation. Younger generations experience second hand their parents’ schooling biographies, aspirations for their children, the schooling system and its consequences for their own lives. Thus the paired voices of mother-father speak about the schooling they have helped their sons and daughters achieve in the locality whilst the younger generation’s voices speak of this continuity of their parental experience, and also show departures from it, as they reflect upon their own schooling experience and talk about their aspirations for the schooling of their own children which reflect but also mediate their own experiences of parental engagements in schooling.

The story, the discursive telling and the dialogical relationship between the two-paired generational narratives tease out meanings and interpretations, and the relationship between social origins and destinations. They shed considerable light on how the narrator is ‘enabled’ or ‘constrained’ in different eras, times and places, and a family’s desire to improve itself despite poverty over generations and over time. One can hear both dispositional effects of social inequality but also micro-social change displayed in the voices of the younger generation brought about through schooling experiences and individual dispositional responses to family history.

**3. *A vertical gender listening***

This listening reveals how gender pairing can illuminate the role of schooling in family decision-making. The aim is to compare the masculine identities, choices and aspirations of the father and son, with the feminine identities, choices and aspirations of the mother and daughter.^10^ In our analysis of the rural habitus, we found in some cases ‘strong patriarchal structures, concepts of honour and shame’. For example, imprisoned in poverty, the women in Rehmat’s family were likely to experience ‘subtle violence of restrictive gender expectations, internalising the religious value of their duties’ (Arnot and Naveed 2014, 521–522). In contrast, women in the upwardly mobile Aslam’s family attained more education, more economic independence, participated in decision-making, and were concerned about self-development (519). In contrast to these two extremes, Akhter’s family attempted a ‘complex reconciliation of the secular and the religious [which] gave the daughter and mother some space within which to increase their self-confidence and be consulted, even if it was not sufficient for them to achieve economic independence’ (Arnot and Naveed 2014, 521).

This gendered listening allows us to develop a structural and dialogic analysis of the *intra-family gender narrative* revealing the moments/patterns of gender congruences but also cross gender negotiation. By attending to the structure of speech and paratextual information, we suggest that comparative educationalists should try to understand the *how* and *why* of the complex gender narratives at play in the family (as well as mapping them). The advantage is that a girl’s education, for example, is not extracted from her family’s dispositional world and the family’s understanding of the economic, social status implications and social mobility opportunities involved. Rather a girl’s schooling is located in the forms of persistence and change associated with what Bourdieu (2001) described as *masculine domination*. This listening reveals the dynamics associated with such domination *within* each generation, along with points of intervention and agency. The *Habitus Listening Guide* captures the more subtle negotiations across gender which shape the individual habitus of family members (particularly those of educated young people) and how it affects the valuing of gender differentiated schooling outcomes. We soon therefore leave behind homogenising views of ‘traditional gender values’ and oppressed silenced mothers (Fennell and Arnot 2008).

The further development of this listening has involved us thinking through how to hear the gendered power relations which are present, both ‘in the order of things’ which represents what is normal and natural, as well as, in the embodied state, the *habitus*. We had already recognised that the gender order was likely to shape the durable dispositions of girls and women (that in ‘lived’ terms result in the embodiment of male over female power structures), shaping aspirations and strategies that reproduce the gender as well as the sexual order. Our revised listening hears how such internalisation of these orders leads to what Bourdieu calls *gentle violence –* a form of *symbolic violence* that is exerted through language and life styles, such as ways of thinking, speaking and acting (Bourdieu 2001, 2). This violence may not always be ‘gentle’ as it were but what it characterises is the framing of gender power relations *within* the ‘understanding’ of the social actors. Our data resonates with the passage where Bourdieu hints at the nuanced naturalness, as it were, of such shaping of the female (dominated) habitus. His argument about the naturalisation processes and their consequences resonated with our initial listenings to the three families:

The dominated apply categories constructed from the point of the view of the dominant to the relations of domination, thus making them appear as natural. This can lead to a kind of systematic self-depreciation, even self-denigration … and, more generally, in their [women’s] adherence to a demeaning image of woman. Symbolic violence is instituted through the adherence that the dominated cannot fail to grant to the dominant (and therefore to the domination) when, to shape her thought of him, and herself, or, rather her thought of her relations with him, she has only cognitive instruments that she shares with him and which, being no more than the embodied form of the relation of domination, cause that relation to appear as natural. (Bourdieu 2001, 35)

A daughter’s experience and understanding of a sexually ordered social order may come through:

… explicit reminders addressed to them by their parents, teachers and peers, themselves endowed with the principles of vision acquired in similar experience of the world, girls internalize in the form of schemes of perception and appreciation not readily accessible to the consciousness, the principles of dominant vision which lead them to find the social order, such as it is, normal or even natural, and in a sense to anticipate their destiny refusing the course or career from which they are anyway excluded and rushing towards those for which they are in any case destined. (Bourdieu 2001, 95)

Bourdieu (2001, 95). argues that powerful emotional violence results from cultural gender narratives that are dominant in a family, resulting perhaps in young women pursuing an anticipated destiny. As educationalists, we need to listen to mothers’ and daughters’ transcripts, to find the ways in ‘which the dominated, often unwittingly, sometimes unwillingly, contribute to their own domination by tacitly accepting the limits imposed, which often take the form of *bodily emotions* – shame, humiliation, timidity, anxiety, guilt – or *passion* and *sentiments* – love, admiration, respect’ (Bourdieu 2001, 38) Indeed, our listening to three daughters suggests precisely such a personalisation of the gender order in which some brothers appear to have even more restrictive views of their sister’s schooling than their fathers (Arnot and Naveed 2014). Bourdieu (2001, 39) illustrates the deep-seatedness of such symbolic violence, which is often expressed passionately by those who are socially dominated, by referring to their relational features such as duty and devotion, and may live on long after the disappearance of their social conditions of production.

Listening to fathers, mothers, and sons, we can hear the centrality of shared marital concerns about the daughter, mediating all other concerns about her life. As Bourdieu (2001, 15) observed, families can be read as institutions of kinship and marriage which assign women a status of the object of exchange serving the interests of male order. With this in mind, our third listening encourages comparative educationalists not to baulk at researching gender power relations linking education to marriage rather than the labour market. In the rural Pakistani family culture, for example, traditionally women are married at very young ages, leaving their family home to live in their husband’s village. They may well find themselves ‘being condemned to circulate as tokens and thus to institute relations between men, they [daughters] are reduced to the status of instruments of production or reproduction of symbolic or social capital’ (Bourdieu 2001, 115). [our addition].

However, our gender listening across generations can also provide evidence of transformative elements within the family’s dispositional world, and of agency and individual negotiations of gendered choices. Marriage might, for example, offer opportunities for social mobility, especially if the husband is educated, economically successful and/or has a higher social status. When using this round of listening, we try to hear the transformative agentic tendencies of women and explore the link of their material conditions with the production of such tendencies (Bourdieu 2001, 40). What gender researchers in development contexts may fail to refer to is how young women aim to achieve the goal of ‘becoming educated’ to increase the prospects for a good marriage, or even literate enough in order to be sufficiently religiously informed and committed to being a good religiously dutiful mother (see the reference to Akhter’s family above). This brings us to the fourth and final listening we have now developed.

**4. *A mythic-ritual listening***

The evolution of the *Habitus Listening Guide* meant recognising and indeed valuing the significance of religious and other organising values in family life*.* A number of families we interviewed in Punjab drew upon religious beliefs to justify educational decisions or their moral stance. Rarely is the centrality of religious beliefs in the lives of the poor included in qualitative data analysis. Yet it is noteworthy that in Kothari and Hulme’s (2004) analysis of the Bangladeshi family, we are told that the mother draws upon ‘specific socio-religious understandings and explanations of her experience’ (15). The authors conclude that ‘these ‘common sense’ understandings and discursive norms construct and embed individual ontologies … . She thus reflects through her testimony the wider context within which the events take place’ (Kothari and Hulme 2004, 33).

Social scientific approaches to qualitative data analysis, which neglect or diminish the power of religious values on family life and forms of socialisation may give the impression that research participants are ‘naked’, secularised, modern subjects – a representation that is inappropriate for societies which are characterised by religious diversity or conflict. Also, where religion is noted, Islamic scholars have found (c.f., Shariati 1986; Zaman 2009) that the approach of mainstream social sciences is rooted in the Western experience of Christianity which is drastically different from the religious experience of other societies. Educational research especially in religiously oriented societies needs to recognise that, as a meaning system, having a religion or religious identity provides an important philosophical orientation that affects individuals’ understanding of the world, making realities and sufferings comprehensible and bearable (Park 2005, 711), while also affecting their subjective and objective wellbeing (Devine, Hinks, and Naveed 2017). Religion can also function as ‘a consecrated basis for prevailing norms and social structure’ and can therefore, encourage ‘the acceptance of the social order and discourages questioning and innovation’ (Schwartz and Huismans 1995, 750).

The most striking features of the voices of rural families we researched were the references to *religion,* the *divine,* and *fate*. As Bourdieu (1991) himself recognised, through his anthropological work amongst the Kayble in Algeria, that what he called the ‘mythic-ritual’ (belief systems that are broader than any specific religion) can be drawn upon to justify, legitimise and hence reproduce the social world. In the context of poverty, religious references might support a particular form of ‘structuration of the perception and thinking of the world’ (Bourdieu 1991) and hence contribute to the maintenance of poverty and social inequality. He argued that religion can have:

an effect of consecration in two ways: (1) It consecrates by converting into limits of law, through its sanctifying sanctions, the economic and political limits and barriers of *fact* and, in particular, by contributing to the *symbolic manipulations of aspirations*, which tends to ensure the adjustment of actual hopes to objective possibilities; (2) It inculcates a system of consecrated practices and representations whose structure (structured) reproduces, in a transfigured and therefore misrecognizable form, the structure of economic and social relations in force in a determinate social formation. (Bourdieu 1991, 14)

The new challenge is for international and comparative research on schooling in such ‘Other’ non-secular contexts, to consider how religious beliefs, in this case Islam, can help members of families define their educational goals and the strategies they need to use to achieve these goals, or how references to religion can add objectivity to subjective relations but also to social relations, thus limiting the possibilities for challenging the ‘conditions of their existence’ (Emmons 2005, 732). In this context, we can take note of how parental religious beliefs can both be used to justify the educational success of their children but can also be used to explain their inability to educate their children (for example by accounting for the lack of good fortune such as the loss of livestock or ill health). The social order can perpetuate itself if the dominated fail to appreciate the arbitrariness of poverty by taking it as natural – the effect of ‘recognition-misrecognition’ (Bourdieu 1991, 14). Our interview data revealed just how important such religious beliefs and identities were, particularly to the poorest of our three families (Arnot and Naveed 2014).

Islamic scholars also understand that, although religion can play these roles in relation to social injustice, it can, for example, also provide the context and incentive to challenge inequality.^11^ Mythic-ritual beliefs and religions offer the incentive and opportunity for individuals’ proactive engagement in challenging the circumstances they find themselves in. For example, such beliefs can be called into play when inspiring strategies to disrupt the power structures. They can give a sense of meaning to life, shape strong education values, and can even associate political and educational struggles against poverty with religious duty.

Using these insights, our fourth listening cautiously offers a way of exploring the level of religiosity which shapes young people’s and their parents’ understanding of schooling and its outcomes. Methodologically, even though we did not directly question interviewees on their religious values, nevertheless we found that some families repeatedly made such references. We therefore extracted from the interview transcripts all (and only) those sentences which refer to religion and religious beliefs. These sentences, following Brown and Gilligan’s ‘notion of an I-poem’, represent what we call a ‘*spiritual poem’* in which we find the intimate ways in which those we interviewed draw upon their spiritual and religious beliefs to understand the social order in which they are positioned, and the ways in which family members’ religious, spiritual and moral outlooks and commitments enter into, shape and reshape their narratives about their own educational histories and that of their children. Even if not religiously active, family members in poor communities may position themselves in relation to the socially and spiritually ordered world in which they live. Those whose social status positions them within such a spiritually ordered world are more likely to consider the interaction between schooling and such beliefs and will be aware of the tensions associated with the ambition to use schooling to be socially mobile within a closed social structure.

This mythic-ritual listening delves deep into people’s dispositional worlds and differentiates between those who explain the possibilities but also limitations they face in their lives, and those who collectively and spiritually seek explanations in their own often individualised agency and practice. The impact of mass schooling with its expectations of individual academic success works upon these different explanatory/legitimatory frameworks used by the poor. In comparative and international education, we cannot leave such religious beliefs at the periphery of our understanding of how families cope with the stresses of living in poverty nor their story about how and why they did or did not educate their children.

## Reflections on the habitus listening guide

The aim of this article has been to contribute to the deepening analysis of the educational values, aspirations and practices of families and young people by developing more sophisticated and deeper listenings to what they can tell us. Takayama, Sriprakash, and Connell (2016) argued that, if we are to break away from colonialist hegemonic models of comparative education (in which other cultures are considered in light of modernisation, Westernisation and ‘progress’), we need to question ‘what we know as well as what we do not know, how we come to know as well as how we come not to know and how we relate to one another in producing comparative knowledge on a planetary scale’ (S19). Various writers such as Green (2006) have recommended a strong qualitative, if not anthropological, perspective to challenge the constitution of poverty as a category of development thinking and as a label which is applied to particular social groups. She argues that poverty is not a measurable condition – rather it is a qualitative social relation.

In light of this, over the last five years, we have developed the *Habitus Listening Guide* as a methodological tool with four different identifiable ways to listen to interview transcripts or other types of narrative. The listenings described here all have the objective of improving understanding of how young people in relation to their family members perceive their education, how they engage with their own schooling and that of their future children. Their narratives provide deeper insights into why some families are able to move out of poverty using schooling, whilst other employ other mechanisms to find better lives, or why some are locked down in acute poverty and despair. The complex historical and contemporary construction of their narrative, their intertwined generational and gender stories, and their references to religious duties or fates, offer new insights into the role of schooling in reproducing or transforming social inequalities. In Table 1 below we summarise the methods we used to ‘hear’ such voices and the objectives of each of our various listenings.

**Table 1.** Listenings, methods and objectives.

Listenings	Methods of voice analysis	Objectives
1. Social structural listening	We listen for the overlapping yet distinctly different descriptions of educational provision and community life, political events, the relationships between various educational events and the relationship of such events to narrators’ values. We conjoin four family members’ narratives to elicit the plots and discursive tools used to connect different parts of the family history/discourse to form a unique whole.	This listening allows us to understand the key structures and objective conditions, the material reality, that surrounds family members’ lives in their community, their relation to government and to the education system. The reconstruction of four interlinked cross-generational biographies, along with images of the social order and social change in the community, differentiates families and their interpretations of their community histories and the effects of education on their community.
2. Horizontal intergenerational listening	This listening elicits a range of dimensions from within and across the paired interviews of husband and wife and the paired interviews of son and daughter – a generational cut that allows comparisons of: the time sequence offered by each set of important events; the role of schooling in generational biographies; the complex interweaving of a range of parental and young people’s narratives; and, the generational impacts of inequalities/poverty.	The dialogical relationship between two generational narratives about poverty, education, aspirations and defeats, sheds light on the place of schooling within the family dynamic and its desire to improve itself. We hear how the narrators’ voices are differentially enabled and constrained in different eras, times and places. We capture the values of parenthood but also filial duties and dependencies.
3. Vertical gender listening	This listening involves: hearing the ways in male identities and duties as parents or siblings are described by the combined messages of father and son, and how fathers and sons’ understandings of their educational duties compare; combining the understandings of mothers and daughters of women’s position, duties and their female negotiating power *vis a vis* husbands and brothers and, the two paired accounts are compared in so far as they construct gendered boundaries within the family, define differential goals for sons and daughters, show how male controls and female agency work within the family and in relation to schooling.	The representations of agency, duty, capability, and future possibilities are important in framing gender relations and educational patterns. The analysis explores the potentially tacit acceptance of social and family controls imposed on adults and youth in each generation – their submissive and transformative agentic tendencies, how the material conditions of their production create such tendencies. The listening reveals the expression of gendered *bodily emotions* that might take the form of ‘shame, humiliation, timidity, anxiety, guilt’ or ‘*passion and sentiments* – love, admiration’, respect (Bourdieu 2001, 38).
4. Mythic-ritual listening	This involves extracting from the transcript only those sentences that refer to mythic-ritual beliefs. These are then put in a list, creating a *spiritual poem*. These sentences can be specific to a particular religion or theology, or they can be broad notions of fate, moral duty, and ethical principles. Each person’s story is thus explored for the contrapuntal voice of mythic discourse and explanations for the family’s situation, perceived successes and failures and for promoting valued actions in relation to education.	The *spiritual poem* reveals how individuals describe their religious, spiritual and moral outlook and commitments, and how these enter into, shape and reshape their narrative about their place in the community, their duties, own educational histories and that of their children. This also displays the dynamic between the parents and children’s beliefs as they mediate the purposes and outcomes of a secular and/or religious education. We can hear generational shifts in the use of mythic-ritual beliefs (in comparison with secular legitimations) to explain, for example, their notions of luck, duty, fate and prospects and how these beliefs affect poverty and/or progress.

Comparative education addresses global agendas around ‘education for all’ by mapping social change using quantitative indicators – yet social change is experiential and often so small as to be seen as insignificant. But small changes are precisely how the family as a group might move forward without disrupting the order which it tries to maintain, even in the face of acute poverty. Social change is not linear – it is erratic, negotiated, resisted, rejected as well as grasped, reflected, internalised and embodied. The introduction of mass schooling, particularly in rural areas, is a massive intervention into a social order. It involves a reordering of the strata within communities and their relations with each other. The new category of the ‘educated’ begins to replace existing social stratifications, creating new vulnerabilities amongst those who were not able to attend school or complete basic schooling. Hulme (2004) argues that we cannot generalise that all data from individual case studies will challenge the perception of the image of the ‘faceless armies of the poor’ (163). However evidence about the reality of their choices, the hierarchy of strategies of survival, and contradictory efforts which might support or undermine their ‘capacity to derive a livelihood’ (171) can show the (hero or villain) role of civil society, markets and the state in the lives of the poor. Such nano-level family-sited research, even more importantly, highlights the absence of thinking about the role of the family – the undervaluing of this site which is difficult to measure quantitatively.

We have argued that establishing the grounds for comparison of the effects of mass schooling involves the collection of experiential, perspectival and dispositional data from families. Merging critical narrative theory with Bourdieu’s cultural reproduction theory and his concept of habitus has allowed us, as researchers, to recognise the structuring impact of poverty and social disadvantage on the lives of families but also the possibilities of hearing the interpretive complexity of lives (Lather 1994). Our Listening guide provides a methodological tool with which to hear the ‘non-linear, poly-vocal multi-levelled’ voices of the poor telling us about their strategic engagements in relation to their values and actions *vis a vis* schooling. Although time costly, the *Habitus Listening Guide* opens up greater understanding of difference, otherness, and diversity within one household. The repayment for the effort of hearing about (at least two) family members’ lives (Camfield and Roelen 2013, 599) reveals their ‘linked lives’ and the educational values associated with parental duty and desires for a good life.

In a context where international development is premised on achieving a global sustainable agenda of giving all children an education, educational researchers need to find ways of not just widening their spheres of actions to other regions of the world, but also of deepening their ability to ‘hear’ what Others might want to tell them. We are aware that the Guide may ‘overread’ (Horsdale 2011) their voices, and that using the powerful tools of Western theory and methodology may need even more deconstructing to be of use in different local cultural contexts. Nevertheless, we believe that such a listening guide is a crucial step towards creating a critically reflexive, decolonising methodological agenda.

## Notes

1. C.f. Connell (2007) for a deep discussion of the need for Southern Theory.

2. Hearing the ‘voices of the poor’ to inform development interventions is not new. ‘Participatory development’, in the 1990s, made the case for development policies to be based on local knowledge and the aspirations, values and priorities of the poor. The three World Bank (2000) reports *Voices of The Poor* represented a major shift by focusing on the perspectives of the poor from 50 countries. The methodological approach shows a tendency to choose a few quotes from the poor placing them appropriately in a narrative that allegedly ‘represented’ their voices without theoretically informed reflection about the processes of generating such voices, and the processes involved in listening, interpreting, engaging with the multiplicity of the meanings of those who spoke.

3. The sampling of youth and their families benefitted from a census of all 477 households in the village. Data from semi-structured interviews with each of the four members of the household were collected during March and June 2010, by a field team consisting of three male and two experienced female researchers including Arif Naveed, all well versed with the Punjabi language. All mothers and fathers agreed to be interviewed and gave permission for their sampled sons and daughters to be interviewed, although not always alone. All female youth were interviewed by female researchers and in some cases, their mother joined the interview. The interviews lasted on average of 90 min, were taped, transcribed and translated into English.

4. Kim’s review of 2029 articles in 8 leading journals which focused on parent school involvement, found that the Western focus on individual achievement and closing achievement gaps neglected the fact that in many developing countries ‘(C)hildren’s educational outcomes were only one of many problems faced by parents who deal with overwork, social instability and are busy securing a livelihood in more precarious circumstances; (7).

5. A ground-breaking longitudinal research programme with 12,000 children growing up in 4 different countries. https://www.younglives.org.uk/.

6. See Annex 1, in Colclough (2012) for details.

7. Manasi Pande’s (2014) listening guide was used to analyse interviews with young carers living in poverty in North India. She explored amongst other elements recurring words, themes, images, metaphors, contradictions, and inconsistencies in style and moral language; she initiated the concept of the ‘We poem’, as a collective agentic notion - a *passive* and *active* ‘We’ in Hindi - in this case an Indian family-oriented voice. ‘The Hindi equivalent of ‘we’ is ‘*hum*’ and is often replaced with ‘*main’* (active ‘I’) to refer to an active ‘I’ in many dialects. In these dialects, when the person uses ‘*hum*’ (we), the context determines whether it refers to the collective ‘we’ or not.’ (ibid.113).

8. See Arnot and Naveed (2014) for a fuller discussion.

9. Reay (2004) argues that empirical research should recognise the lack of action of actors and the ‘pre-reflective’ aspects of their action. Without such recognition, Bourdieu is unfairly criticised for overplaying ‘the unconscious impulses’ and ‘neglecting mundane everyday reflexivity’.

10. Ideally we could investigate cross-gender relations between fathers and daughters, and mothers and sons.

11. Islamic theology places a great emphasis upon poverty eradication. Shariati (1986) argued that the responsibility of the ‘enlightened’ is to respect religious values but also to engage critically with the challenges posed by the socially reproductive forms of religion.
